# Efficient tactile encoding of object slippage

**DOI:** 10.1038/s41598-022-16938-1

**Published:** 2022-08-01

**Authors:** Laurence Willemet, Nicolas Huloux, Michaël Wiertlewski

**Affiliations:** 1 Aix-Marseille University, CNRS, ISM, Marseille, France; 2 Delft University of Technology, Delft, The Netherlands

**Keywords:** Information theory and computation, Motor control, Sensorimotor processing

## Abstract

When grasping objects, we rely on our sense of touch to adjust our grip and react against external perturbations. Less than 200 ms after an unexpected event, the sensorimotor system is able to process tactile information to deduce the frictional strength of the contact and to react accordingly. Given that roughly 1,300 afferents innervate the fingertips, it is unclear how the nervous system can process such a large influx of data in a sufficiently short time span. In this study, we measured the deformation of the skin during the initial stages of incipient sliding for a wide range of frictional conditions. We show that the dominant patterns of deformation are sufficient to estimate the distance between the frictional force and the frictional strength of the contact. From these stereotypical patterns, a classifier can predict if an object is about to slide during the initial stages of incipient slip. The prediction is robust to the actual value of the interfacial friction, showing sensory invariance. These results suggest the existence of a possible compact set of bases that we call *Eigenstrains*. These Eigenstrains are a potential mechanism to rapidly decode the margin from full slip from the tactile information contained in the deformation of the skin. Our findings suggest that only 6 of these Eigenstrains are necessary to classify whether the object is firmly stuck to the fingers or is close to slipping away. These findings give clues about the tactile regulation of grasp and the insights are directly applicable to the design of robotic grippers and prosthetics that rapidly react to external perturbations.

## Introduction

**Figure 1:**
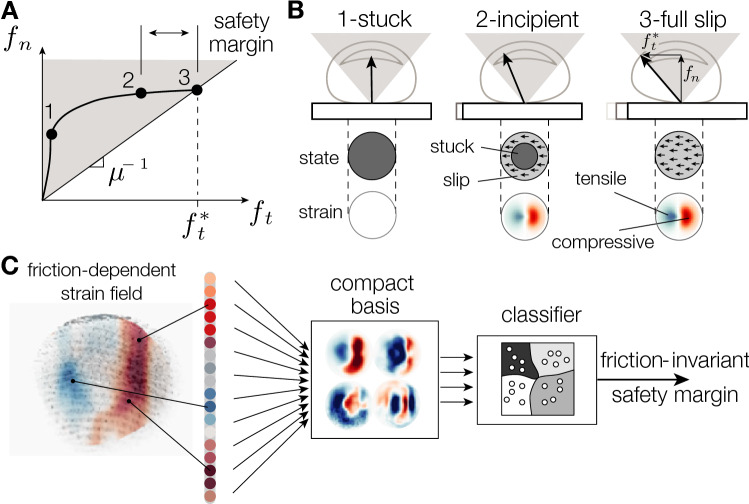
(**A**) Normal and tangential components of the force during grasping. To avoid slippage, the nervous system regulates the grasping force $f_n$ to keep the frictional strength $f_t = \mu \,f_n$ at a safety margin from the load force applied by the object. (**B**) Typical evolution of the interaction force, area of contact, and skin deformation during the transition from stick to slip. (**C**) Steps of perceptual computation from a strain field that is influenced by the frictional interaction to an estimate of the safety margin which is independent of friction.

Dexterous tasks, such as picking fruits or writing with a pen, continuously recruit sensorimotor feedback to detect and avoid object slippage. The feedback loop depends on the information provided by cutaneous afferents. Using this information, the sensorimotor system keeps the object stable in hand while applying a gentle grasp, by continuously adjusting the magnitude of the grip forces. During grip adjustment, a margin of safety between the frictional strength of the contact (i.e. the maximum admissible lateral force before slippage) and the external load forces acting on the object is maintained^1^. The safety margin sets the grip force 10% to 20% higher than the minimum admissible force, depending on the unpredictability of the forces at play^2^. The low safety margin restricts the deployment of large grip forces responsible for muscular fatigue, for damaging fragile objects, and for impeding the reorientation of an object during in-hand manipulation. The typical evolution of the forces during grasping an object is shown in Fig. 1A.

To maintain a safety margin, tactile afferents encoding the spatio-temporal deformation of the skin are continuously monitored^3^. The most convincing evidence is that participants whose sense of touch has been locally anesthetized show a drastic degradation of the dexterous movements and grip force regulation^4,5^. These grip force adjustments are likely triggered by early signs of incipient slippage of the object in contact with the skin. At a mechanical level, during incipient slippage, the contact transitions from a stable state where it is completely stuck, to an intermediate state where the outer region of the contact slips. The slip region grows to eventually encompass the entire contact area, at which stage the stuck area vanishes and the object fully slips^6^. The incipient slip transition, predicted by Cattaneo-Mindlin theory^7^ and illustrated in Fig. 1B, induces stereotypical patterns of skin deformation where tissues are compressed at the leading edge and stretched at the trailing edge^8^. The patterns of strain in turn cause a stereotypical activity of the mechanoreceptors^9^.

Reacting quickly to incipient slip requires processing signals sent by more than a thousand afferents on average^10^ to detect a specific pattern in the spatio-temporal deformation. The deformation depends on the friction of the surface, but since the safety margin is independent of friction^11^, the detection has to be invariant to the friction coefficient. In other terms, the regulation should operate similarly whether the object is slippery or not. Given the complexity of the task, how can the nervous system efficiently process tactile information to quickly detect slip and avoid a catastrophic loss of grip?

In this article, we hypothesize that the nervous system must compress the peripheral information by projecting it on a compact basis of functions as illustrated in Fig. 1C. The compression removes the redundancy and promotes perceptual invariance to friction when detecting incipient slippage. To test our hypothesis, we extracted a compact dictionary of deformation patterns from a large dataset containing the spatio-temporal evolution of skin strains during the transition from stick to slip at different frictional conditions. The dictionary efficiently decodes the safety margin from the pattern of strain with a success rate higher than 80% compared to 20% when using the entire strain pattern. The results reveal the contribution of skin mechanics to the detection of incipient slip and can inspire reactive control of robotic grippers based on tactile events^12,13^.

### Encoding of object slippage

At the onset of sliding, the deformation of the skin likely stimulates mechanoreceptors whose neural activity propagates toward the central nervous system^9,14,15^. The timing and the number of the first spikes of neural activity produced by the deformation contain crucial information, for stabilizing grasp^16,17^. External perturbations elicit responses within 100 to 150 ms^18,19^ during which central processing only accounts for approximately 15 ms of the total time^17^. The latency is comparable in magnitude to long-latency reflex responses, suggesting that the grip force regulation is mediated supra-spinally^20^.

Given the speed of the reaction, the number of stimulated mechanoreceptors, and the limited capacity of the brain, the nervous system likely compresses the information contained in the afferents. One possible compression mechanism involves projecting the incoming skin deformation pattern onto a *compact* dictionary of strain primitives. With the dictionary, the high-dimensional space of the neural information from upward of 1,000 afferents in the fingertip is reduced down to a few principal components. By focusing on a few principal components, a simple set of neurons can provide a swift estimation of the safety margin and determine if more grip force should be applied (Fig. 1C).

### Efficient coding hypothesis

The dimensionality reduction process proposed in this paper derives from the efficient coding hypothesis, first introduced by^21^. Efficient coding postulates that information is transmitted from the sensory organs to the nervous system with a minimal number of action potentials, using a *compact* lexicon that minimizes the neural activity by removing the information redundancy. Moreover, the lexicon must be independent of the friction coefficient, since the same reflexive behavior can be observed on objects having surfaces of various frictional strengths^22^.

How can we gain access to a likely candidate of a compact lexicon? Considering that the sensory system evolves in the natural world, a representation must be created where natural stimuli are encoded efficiently^23^. Therefore, by distilling the lexicon from a large sample of natural stimuli, we can find a compact function decomposition by maximizing the sparsity of the signal. The sparsity assumption allows us to extract useful patterns from big datasets and, thus, reduce the computational cost. In the specific case of detecting incipient slippage, these stimuli are the strain patterns, representative of the deformation of the skin. Similar dimensionality reduction approaches have been successful in distilling sparse representations of natural images^24^ and audio signals^25^. The sparsity condition ensures that the information is embedded in a population code with a minimum number of neurons active at any one time, leading to a more than 20-fold compression of images or audio waveforms without losing perceptual accuracy^26^. Similar efficient coding strategies have been proposed in touch, and can facilitate the classification of hand gestures from vibrotactile surface wave propagation^27^ or to identify material properties from the vibrotactile signal they produce^28^.

### Rationale behind the dimensionality reduction

Amongst the numerous dimensionality reduction methods, matrix factorization methods can efficiently find a dictionary to compress natural stimuli. For instance, independent component analysis finds features separating the signal in statistically independent parts. When applied to natural images, it recovers a functional basis that resembles Gabor filters^23^, hinting at a possible structure of the computation used in the early stages of the visual processing. Similarly, Non-negative Matrix Factorization^29^ has been popular for explaining sensory processing since it promotes basis functions that capture local features. As an example, the factorization trained on a database containing human faces leads to a dictionary containing representations of the mouth and the nose.

In our specific case of decoding the safety margin from the skin deformation, we postulate that the nervous system uses a compact set of basis patterns (i.e. that includes only a minimal amount of projective axes) to accelerate the processing. The compact set of bases should capture the most variance of the skin deformation patterns and should maximally decorrelate the output signal. This set of requirements makes the principal component analysis the most suited method. The principal component analysis can be computed by taking the singular value decomposition (SVD) of the entire database of strain patterns and truncating the result to conserve only the first most representative principal components^30,31^.

## Results

### Dataset of spatio-temporal strain field

We computed the spatio-temporal deformation of participants’ skin while they touched a plate that slid under their index fingertip. We collected the temporal evolution of the strain pattern of the index fingertip of 12 participants, using 7 levels of frictional conditions and 4 repetitions, resulting in 336 individual videos. We selected 30 equally spaced frames of these videos from 0.05 to 6 mm every 0.2 mm of relative displacement between the plate and the finger, totaling in 10,080 data points.

**Figure 2:**
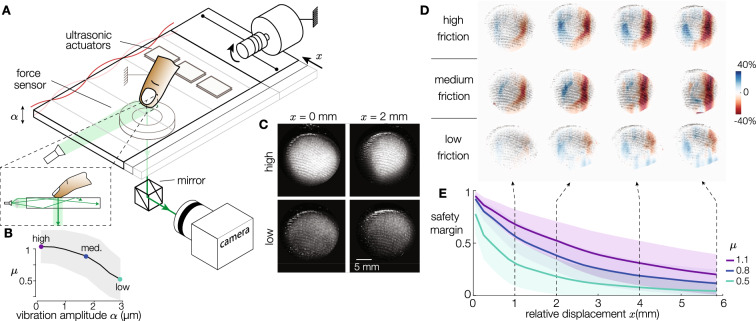
(**A**) Experimental apparatus combining ultrasonic friction reduction and frustrated total internal reflection imaging of the contact (inset). (**B**) Ultrasonic vibration reduces the sliding friction coefficient. (**C**) Typical images for two lateral deformations and two amplitudes of vibrations. (**D**) Experimental deformation of the skin when the finger is sliding on the surface in high, medium, and low-friction conditions for relative displacements of 1, 2, 4, and 6 mm. (**E**) Safety margin as a function of the finger position for the same 3 friction conditions. The solid lines and shaded areas stand for mean ± std.

The friction of the plate could be changed from high, medium, and low friction using ultrasonic friction modulation^32^. The three conditions correspond to average coefficients of sliding friction of 1.1, 0.8, and 0.5, for vibration amplitudes of 0.17, 1.6, and 2.9 $\upmu {\mathrm {m}}$ respectively (Fig. 2B). A constant normal force of about 1.2 N was maintained by a lever and weights, and we imposed the lateral force by controlling the current in a coreless motor, through a low-friction capstan transmission. The plate moved in the radial direction with a speed of 10 mm/s, for a total displacement of 20 mm, which is sufficiently long for the finger to reach full slip. Forces and position are plotted in Figure S1.

The deformation of the skin was extracted from the images of the contact illuminated by frustrated total internal reflection (FTIR). The illumination technique highlights the asperities of the skin that are in intimate contact with the plate while darkening everything that is not touching the plate, resulting in a highly contrasted image^33^. An illustration of the apparatus can be found in Fig. 2A and typical images for a high- and low-friction case are shown in Fig. 2C. Construction details are presented in the Materials and Methods section. The motion of individual points on the surface of the skin was tracked. The skin strains were computed from the displacements of each tracked point using the Delaunay triangulation method^8^, the procedure is depicted in Movie M1. From the start of plate motion and until full slip is reached, the finger experiences longitudinal strains, whose magnitude depends on the frictional strength of the contact as shown in Fig. 2D.

For each element of the dataset, the spatial strain field of the fingertip was matched to the safety margin $S_m$. First, the static friction limit $f_t^*$ was identified from the time series of the lateral force by considering the average force when the finger was fully sliding. Then, the safety margin was computed for all instants in time (Fig. 2E) from:

1 $$\begin{aligned} S_m(t) = \frac{f_t^*-f_t(t)}{f_t^*} \end{aligned}$$

### Empirical strain patterns

**Figure 3:**
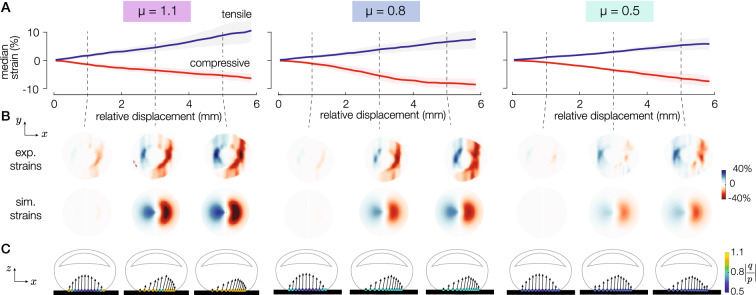
Typical trial. (**A**) Compressive and tensile strain fields for 3 different friction conditions as a function of the relative lateral displacement. The solid lines and the shaded areas represent the mean ± std. (**B**) Experimental and simulated strain profiles for the 3 different friction conditions at three positions on the plate from 1 to 5 mm. (**C**) The corresponding simulated surface finger profile. The blue arrows represent the pressure *p* and traction *q* acting on each element, and the color of the dots represents the local pressure ratio |*q*/*p*|.

During the transition from stick to slip, the finger deforms and the slip area propagates from the periphery to the center of the contact area. The strain wave is always compressive ahead of the stuck area (red in figures) and tensile on the trailing edge (blue in figures), see Figure S2, consistent with previous observations^8^. The strain fields are shown for 3 coefficients of friction of 1.1, 0.8, and 0.5 (Fig. 3B). For all friction conditions, as the plate displacement increases, the magnitude of the tensile and compressive strains increases (Fig. 3A). The magnitude of the compressive strain decreases significantly with increasing vibration amplitudes (ANOVA, F(6,329)=2.18, $p=0.045$), whereas the magnitude of the tensile strain increases with increasing vibration amplitudes (ANOVA, F(6,328)=6.3, $p=0.0091$), see Figure S3. For a high-friction condition, the maximum compressive strain experienced by the finger is on average 25% larger than when friction is low.

### Model validation

To better understand the influence of friction on the skin deformation during sliding, we simulated the interaction using a finite-difference time-domain model that captures the viscoelasticity of the stratum corneum and soft cutaneous tissues as well as the local frictional behavior. The details of the implementation are presented in the Supplementary Figure S4. The fingertip model is composed of a chain of massless elements linked together by high-stiffness springs ($2.5~{\mathrm {kN\,m}}{^{-1}}$). The chain lies on a bed of soft springs ($31.5~{\mathrm {N\,m}}{^{-1}}$) connected on the other end to a rigid element modeling the bone. To maintain contact and induce a sliding motion, external normal and tangential forces were applied to the bone element $f_n = f_t=1~{\mathrm {N}}$. The simulated deformation fields of the skin are shown in Fig. 3B. The simulated strain fields follow a similar trend as the experimental ones, with a compressive part ahead of the stuck area and a dilatation behind it.

The fingertip model allows us to observe the pressure and traction fields at the interface between the skin and the surface that cannot be accessed by experimental means, see Fig. 3C. During the transition from stick to slip, we observe that the elements on the outer edge are the first to slide since the interaction pressure is collinear with the friction cone. In the high friction condition, the lateral motion of the elements is constrained, resulting in a larger skin strain. Conversely, in the low friction condition, the outside layer experiences lower tangential traction, and the lateral stress is released for smaller lateral displacement.

### Dimensionality reduction of the strain field

We postulate that the strain field must contain information about the safety margin before slippage. Since the estimate of the safety margin exists before gross sliding occurs, the estimate is likely independent of the actual friction coefficient of the surface. While we do not have access to the neural encoding of the afferent to find a base of neuronal activation, we can infer it from the skin displacement. The dataset allows us to find a potential basis composed of a set of strain field patterns.

To find the *Eigenstrain* patterns, we performed a Singular Value Decomposition (SVD) of the 10,080 individual strain patterns contained in the dataset. The method outputs a set of orthogonal eigenvectors $u_i(x)$ representing the dictionary of strain patterns, and eigenvalues $\sigma _i$, whose magnitude relates to the variance explained. The weight of each Eigenstrain as a function of time is embedded in *v*(*t*), such that each vector $v_i$ reveals the temporal evolution of the $i^{th}$ eigenvectors. To compress the information, we selected the first *r* elements of the set. The original evolution of the skin strain can be recovered by adding these eigenvectors, weighted by time-dependent vectors, ${\sigma _i} v_i(t)$ as follows.

2 $$\begin{aligned} \hat{\epsilon }(x,t) = \sum _{i=1}^{r} u_i(x) {\sigma _i} v_i(t) \end{aligned}$$

**Figure 4:**
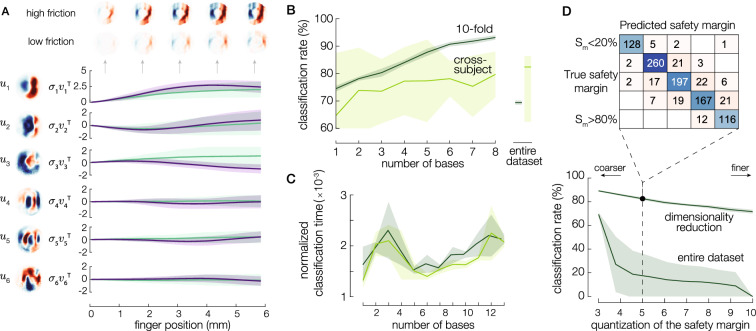
(**A**) Six first bases $u_i$ and the temporal evolution of the recruitment of these six bases for a low and a high-friction condition (in violet and green, respectively). (**B**) The classification rate of the safety margin is split into two classes, as a function of the number of bases. The dark green line corresponds to ten-fold testing when taking 90% of the data for training and 10% for testing, and the light green line corresponds to cross-subject testing when only one subject among 14 is used for testing. The solid lines and shaded areas stand for mean ± std. Classification rates using the entire strain matrix $\epsilon$ are represented on the right. (**C**) The time needed for the classification using $\Sigma V$ normalized by the time using the whole matrix of strains for the cross-subject and the 10-fold classifier. (**D**) Effect of safety margin quantization on the classification rate when using 6 bases for the decomposition. The confusion matrix is shown for 5 classes.

The first six primitives are shown in Fig. 4A. $u_1$ is the major principal component, illustrating the typical pattern of compression ahead of the stuck area and stretching behind it. $u_2$ and $u_3$ include higher frequency details at the periphery of the contact, whereas the following bases improve the details at the center of the contact area.

Figure 4A shows the recruitment of each basis ${\sigma _i} v_i^T$ as a function of time, for a high and a low friction coefficient. The recruitment of the first basis differs between high and low friction conditions from the early stages of the slip when the finger has moved 0.25 mm relative to the plate (Spearman’s correlation, $\rho =-0.17$, $p=0.0024$). The amplitude of the first basis captures the intensity of the skin deformation. On the other hand, the recruitment of bases 2, 4, 5, and 6 are not significantly impacted by the level of friction. Similarly, the recruitment of the third basis changes significantly with friction when the relative displacement is higher than 1 mm (Spearman’s correlation, $\rho =-0.21$, $p=8.4\times 10^{-5}$). ${\sigma _2} v_2^T$ do not significantly differ between the friction conditions, suggesting that the friction does not significantly influence the tensile pattern embedded in $u_2$.

### Efficiency of the tactile encoding

We trained two support-vector machine classifiers to predict the safety margin from the recruitment of the bases ${\sigma }v^T$. The first one was trained using 90% of the whole dataset (10-fold) and the second one with data of the whole subjects, except one which was used for testing (cross-subject). The prediction map using the first two bases with the 10-fold classifier is shown in Figure S7A for 2 classes of safety margin: higher and lower than 0.5.

Compressing the tactile information with only 2 bases leads to a classification rate of 70%, whereas this number increases with the number of bases and exceeds 90% of accuracy for 6 bases (see Fig. 4B). Adding more than 6 bases leads to a marginal increase in the classification rates, and the performance of the 10-fold classifier drops when using the entire dataset. The classification rates for cross-subject classifiers are lower and present larger standard deviations than the one with a ten-fold training, due to the high inter-subject variability.

Since humans react in a remarkably short amount of time, we qualitatively compared the speed of each classification approach, by studying the influence of the number of bases on the computational effort. To get a qualitative estimate of the computational effort, we computed the time needed for the cross-subject and 10-fold classifiers to perform the prediction, normalized by the time of classification using the whole matrix of strains (Fig. 4C). For both classifiers, the predictions using a limited number of bases are performed more than 600 times faster than using the entire strain data; the latter takes around 23 s. Moreover, the relative classification time between the limited number of bases and the entire strain matrix is minimum when considering only 6 bases. The minimum of computational effort suggests that the 6-bases kernel provides an efficient estimation while preserving accuracy. This value matches the tradeoff between precision and compactness of the bases (Figure S6B): selecting less than 6 bases lacks accuracy, whereas considering more than 6 bases leads to a recruitment matrix *V* not compact enough, which is less efficient to process.

To increase the accuracy of safety margin estimates, we reduced the interval quantization of the safety margin by increasing the number of classes from 3 to 10 (Fig. 4D). The classification rate using 6 bases decreases when the number of classes increases, but stays higher than 70% even when the safety margin was predicted with a 0.1-precision using 10 classes. Increasing the discretization of the safety margin comes with a significant tradeoff in the classification rate.

We also studied the influence of adding short-term memory to the classifier. We trained the classifier with knowledge of the short-term evolution of the recruitment on each basis. We find that the accuracy of the safety margin estimation using the 10-fold classifier trained with the contribution of the 6 bases at a given time instant was 20% higher than using the contribution of the first basis at 6 consecutive instants. However, adding priors on the weight of the first and second bases increases the accuracy of the cross-subject classifier by 10%, in comparison with exclusively spatial or exclusively temporal values (Figure S7C).

## Discussion

The findings suggest the existence of compression of the tactile information of incipient slippage, showing one concrete implementation of the idea that the computation behind tactile perception is embedded in a minimal subspace^34^. The six strain primitives obtained with the singular value decomposition enable a reduction of the dimensionality of the tactile signal while keeping a sufficient accuracy of the predictions. We found a major contribution of the compressing strain in the encoding of friction, which has recently been shown to excite the response of fast adapting afferents of type 1 (FA-I)^22^.

The first six bases were found to optimally encode the safety margin, leading to a 90% accuracy of the safety margin quantized over two classes. When the safety margin was quantized with more than seven classes, the accuracy decreased to 85%. Globally, if the number of bases exceeds the number of classes, the classification rate is higher than 80%. However, when reacting to an excessive reduction of the safety margin, the sensorimotor system is likely to make a binary decision. The decision could be based on a quantization of the safety margin that involves only two classes and therefore a limited number of bases.

We estimated the safety margin at specific time stamps, without taking into account the history of the deformation that led to a particular strain pattern. Knowledge of the dynamics could help improve the prediction of imminent slippage. Since the adjustment of the grip force is a continuous process, it is likely that the nervous system constantly monitors the time differences in strain to make a judgment. Assuming that the detection of slippage makes use of predictive coding, the evolution of the strain could be associated with priors on the weight and material property to lead to an even more robust classification^35^. The classification rate of the 10-fold classifier is 10% lower when the prediction is made with exclusively temporal evolution of the first basis compared to a purely spatial one. Future investigations will include several scanning speeds to properly study the influence of the skin dynamics on the classification of the safety margin.

It is worth noting that the mechanics dictating the skin deformation is strongly influenced by the friction of the surface. Large friction coefficients lead to large compressive strain of the skin, in line with previous findings. The strain profiles observed when the finger is sliding on a friction-modulated glass plate matched with the previous one observed in the literature with a slip annulus forming at the periphery first^6,8,36^. The classifier successfully removes the dependence on friction, suggesting that the information of the safety margin is contained not in the magnitude of the strain, which is strongly influenced by friction, but in the relative recruitment of the different Eigenstrains.

In this study, the database is constituted with data acquired in constrained conditions when the plate is moving in the ulnar direction to mimic an object slippage due to gravity. Since it is known that the direction of the slippage has a significant influence on the strain experienced by the finger^8^, different orientations might likely be encoded in the nervous system (see Figure S6C). We carefully aligned participants’ index finger to have a consistent center of the contact across the dataset, necessary to avoid alignment artifacts when using the singular value decomposition. Since the center of contact varies constantly during natural interaction, it is likely that the nervous system uses a process analogous to a convolution rather than a simple projection to ensure the translation-invariance. This study was limited to a perfectly flat and smooth glass plate. The actual set of bases probably incorporates an invariance to the physical properties of the object, such as local texture and curvature that would affect the shape of the strain field.

Interestingly, the optimal basis of strain patterns resembles a collection of Gabor filters, containing alternative patterns of compression and tension. While the first basis has only one cycle of alternating strains, the higher-order pattern contains a higher frequency feature that captures finer details of the interaction. It has been hypothesized that a bank of Gabor filters is used to encode tactile features^37^. These filters are central to the perception of movement in the visual system, and their presence in the tactile perceptual system suggests that their function is shared across modalities.

The corresponding temporal evolutions of the recruitment of each of the six bases, compactly represent the evolution of the strain field. By virtue of its compactness, the code simplifies and accelerates the decoding by the nervous system, which is needed to react promptly while avoiding slippage of an object in hand. Even if the existence of a compact lexicon in the human nervous system still needs to be confirmed, the *Eigenstrain* decomposition can be directly used to design efficient control policies for robotic grippers that can manipulate objects while preventing slippage instead of reacting to it^38–40^.

## Materials and methods

### Data collection

Twelve volunteers gave their informed consent prior to the experiment to participate in the study, which was approved by the institutional review boards of Aix-Marseille Université’s ethics committee (2019-14-11-003). All methods were performed in accordance with the relevant guidelines and regulations. The index fingertip of the participants was secured in a dedicated 3D printed plastic shell to ensure a constant angle between the finger and the glass plate around 20$^{\circ }$. The frictional resistance of the plate against the skin was controlled by ultrasonic lubrication^32^. The device uses a flexural standing wave to induce a micrometric levitation of the skin of the fingertip, thereby reducing the interfacial friction. The rectangular glass plate vibrated at a frequency of 29.97 kHz in the $3 \times 0$ mode, $68\times 120\times 11~{\mathrm {mm}}^3$. Images of the fingertip were captured at 300 frames per second by a high-speed camera (Phantom Miro M110). Frustrated Total Internal Reflection (F.T.I.R) was used to highlight the asperities of the skin in intimate contact with the glass plate. The technique creates highly contrasted images of the skin asperities at pixel resolution, that is 0.0535 mm.

The haptic surface is mounted onto an aluminum frame attached to a 6-axis force sensor (ATI Nano 43). The normal force applied to the finger was controlled with a balance mechanism, with one arm pushing against the finger and the other arm supporting a calibrated weight. The lateral force developed was servo-controlled by a DC-motor (Maxon RE 36) with a capstan transmission.

### Data analyses

Force data was synchronized to the images using a digital trigger also used to start the movement. The time-domain data were interpolated to match the time vector of the images. The force data were filtered using a zero-lag 50 Hz second-order low-pass filter. For a good measure of plate displacement, a checkerboard pattern was printed on the glass plate to get an external reference of the relative motion.

The contrast of the image was adjusted, and the contour was sharpened. 3000 optimal features were selected within a fitted ellipse of contact, extracted from the binarized image. The selected features were nearly equally spaced with a minimum spacing of 10 pixels, to be sure the entire population of features is equally distributed inside the ellipse of contact. Then, these features were tracked frame by frame with sub-pixel accuracy. The relative displacement of each feature was obtained by subtracting its current position from the initial value found before the movement started.

The longitudinal strain fields were obtained via the same procedure as in^8^, using the equation:

3 $$\begin{aligned} \epsilon _{xx}&= \frac{\partial u}{\partial x} + 0.5 \left[ \left( \frac{\partial u}{\partial x}\right) ^2 + \left( \frac{\partial v}{\partial x}\right) ^2 \right] \end{aligned}$$

Each strain field was interpolated on a grid of $601\times 801$ pixels and downsampled 8 times, leading to a $76\times 101$ matrix. Skin strain was not resized according to the finger size to promote diversity in the dataset. Since the safety margin depends on the size of the fingertip, including the strain without rescaling leads to better classification.

### Efficient encoding

The optimization procedure used for dimensionality reduction was formulated as a Limited Memory Block Krylov Subspace Optimization, allowing to maximize the compactness and the accuracy of the estimation^41^. For each value of the rank *r*, the optimal set of bases was determined by minimizing the difference between the strain and its estimate.

## Supplementary Information

Supplementary Information 1.

Supplementary Information 2.

Supplementary Information 3.

Supplementary Information 4.

Supplementary Information 5.

Supplementary Information 6.

Supplementary Information 7.

Supplementary Information 8.

Supplementary Video 1.

## Data Availability

The datasets generated and/or analyzed during the current study are available in the 4TU.ResearchData repository, https://doi.org/10.4121/19329506.v1.
