# Supplementary figures and images for: Efficient tactile encoding of object slippage
Source: Sci Rep. 2022 Aug 1;12:13192. doi: 10.1038/s41598-022-16938-1 (PMC9343352; doi:10.1038/s41598-022-16938-1)

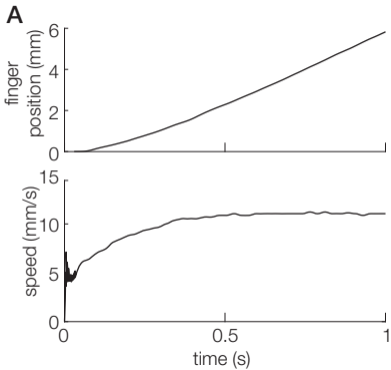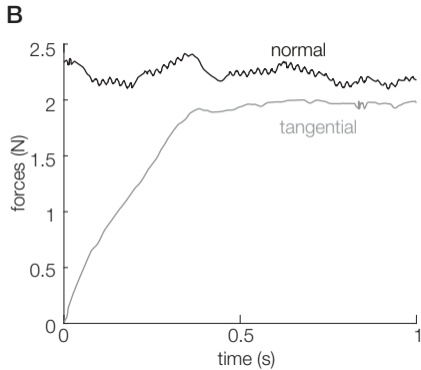

Supplement: Supplementary file 1 — Supplementary Information 1. [file 41598_2022_16938_MOESM1_ESM.pdf]

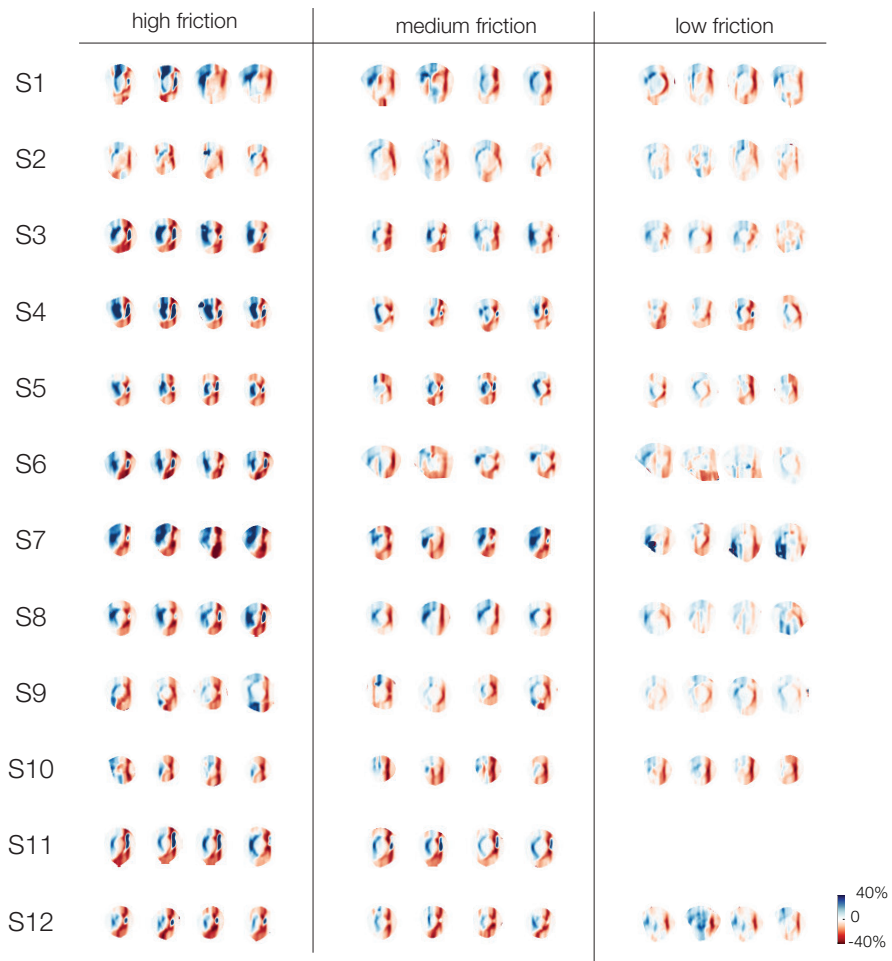

Supplement: Supplementary file 2 — Supplementary Information 2. [file 41598_2022_16938_MOESM2_ESM.pdf]

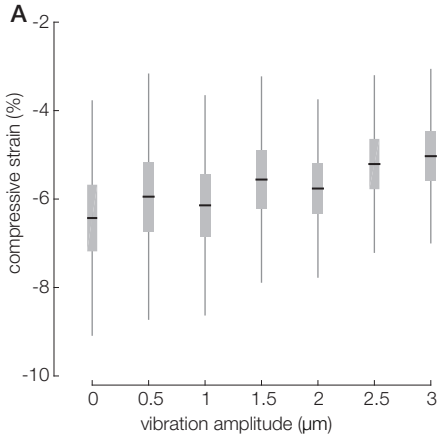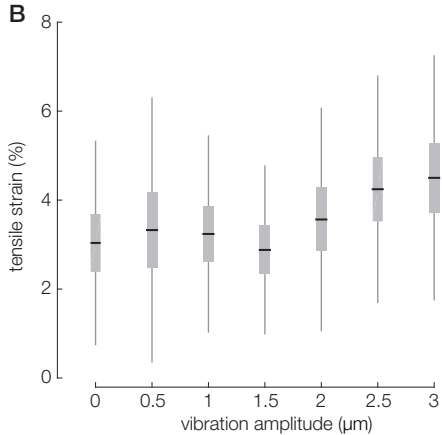

Supplement: Supplementary file 3 — Supplementary Information 3. [file 41598_2022_16938_MOESM3_ESM.pdf]

**A**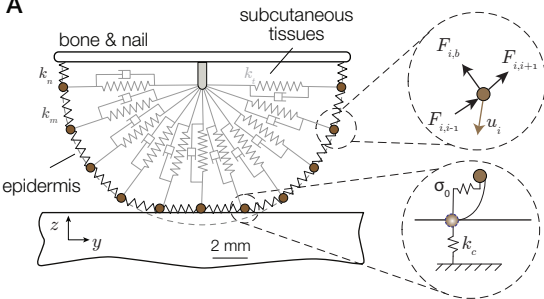**B**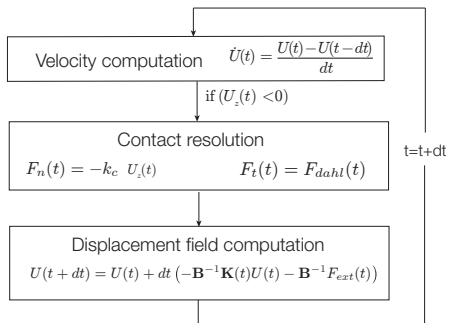**C**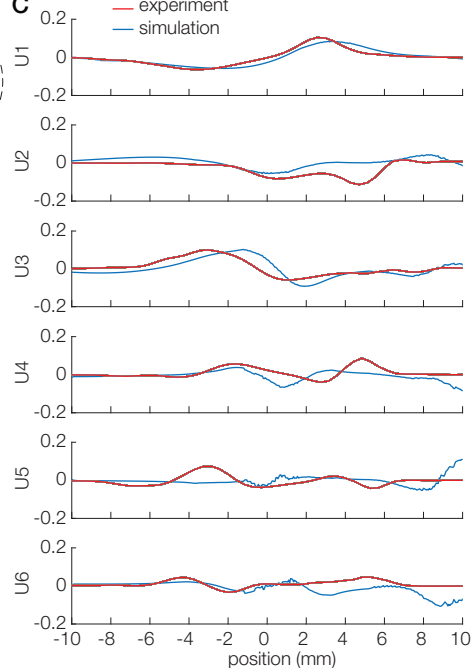

Supplement: Supplementary file 4 — Supplementary Information 4. [file 41598_2022_16938_MOESM4_ESM.pdf]

**SVD** $t_c = 1.62$  s $\Delta = -3.3e^{-3}$ 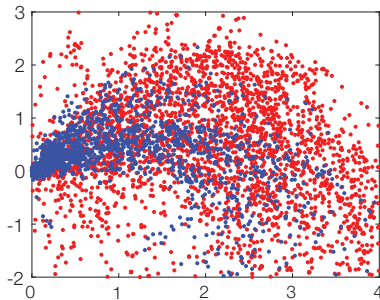**ICA** $t_c = 57.3$  s $\Delta = -2.68e^{-5}$ 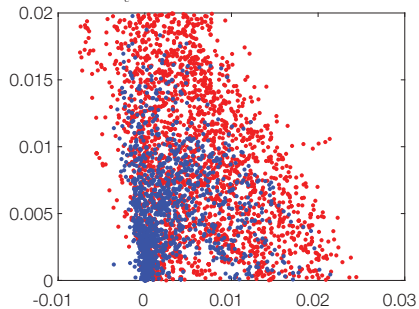**NMF** $t_c = 11.2$  s $\Delta = -5.7e^{-3}$ 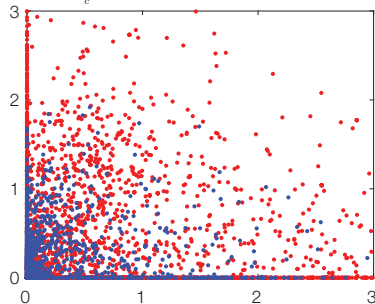**t-SNE** $t_c = 928$  s $\Delta = -0.52$ 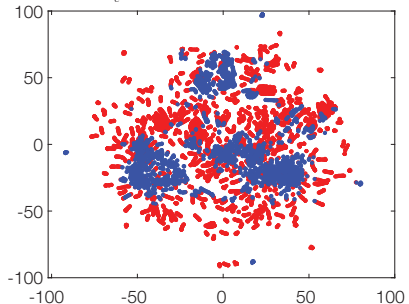

•  $S_m < 0.5$   
•  $S_m > 0.5$

Supplement: Supplementary file 5 — Supplementary Information 5. [file 41598_2022_16938_MOESM5_ESM.pdf]

**A**  $\mu = 1.1$   $\mu = 0.8$   $\mu = 0.5$

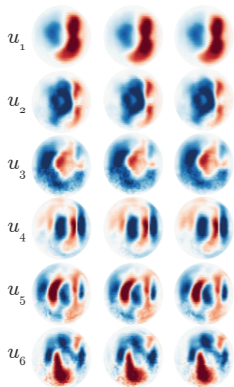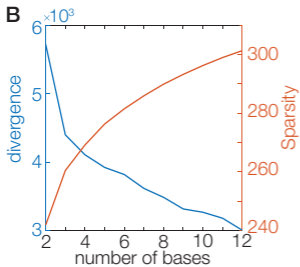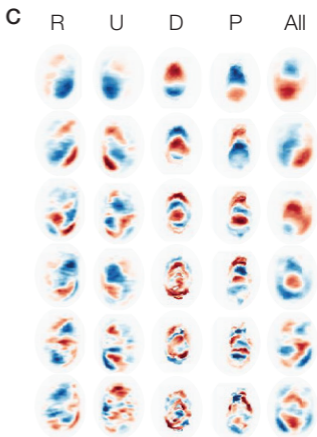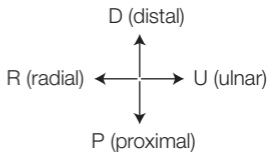

Supplement: Supplementary file 6 — Supplementary Information 6. [file 41598_2022_16938_MOESM6_ESM.pdf]

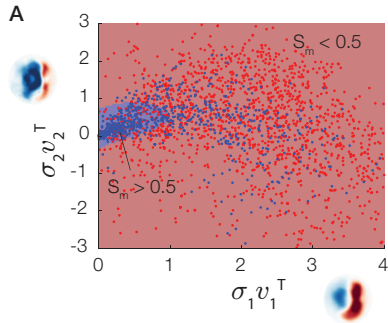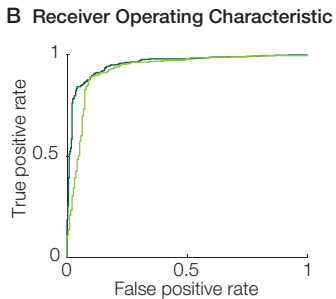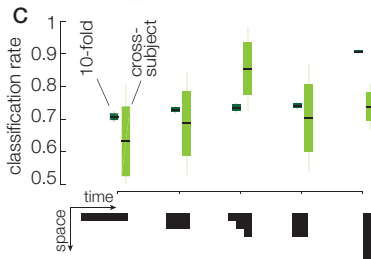

Supplement: Supplementary file 7 — Supplementary Information 7. [file 41598_2022_16938_MOESM7_ESM.pdf]
