# Supplementary material for: Efficient tactile encoding of object slippage
Source: Sci Rep. 2022 Aug 1;12:13192. doi: 10.1038/s41598-022-16938-1 (PMC9343352; doi:10.1038/s41598-022-16938-1)
Supplement: Supplementary file 8 — Supplementary Information 8. [file 41598_2022_16938_MOESM8_ESM.pdf]

Supporting information

Laurence Willemet, Nicolas Huloux and Michaël Wiertlewski

July 6, 2022

Supplementary text

1 Dataset acquisition

We acquired images of the fingertip when the plate slid in the radial direction from 0.05 to 6 mm every 0.2 mm of relative position between the finger and the plate during the slippage. Forces and positions are plotted on Figure S1.

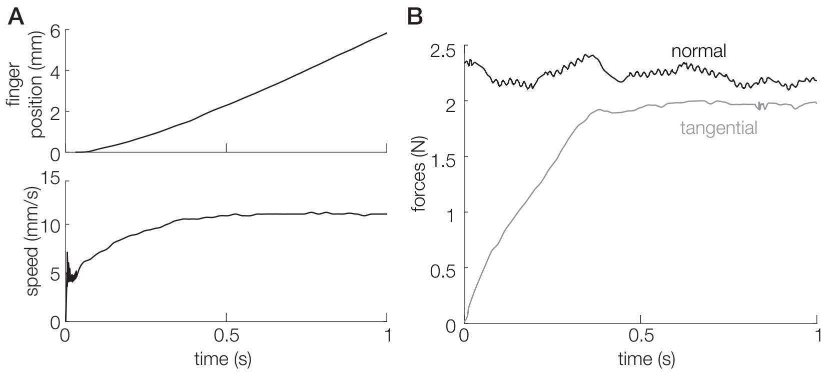

Figure S1: **A**. Time series of finger position and speed respectively. **B**. Normal and tangential forces applied by the finger.

The longitudinal strain components ϵ_{xx} were computed using 3000 tracked points. Then, the strains were interpolated on a grid of size 601×801, and downsampled 8 times, resulting in 76×101 matrices.

The data set of skin strains was represented by a $m \times n$ matrix, $\mathbf{X}$, where m is the number of spatial positions at which the strain is interpolated (7,676) and n is the number of recorded trials times the number of relative positions between the finger and the plate at which the strain is computed (10,080).

Four skin strain data for each friction condition (high, medium, low) and each subject are presented in Figure S2. For subject 11, the ultrasonic vibrations cause a limited friction reduction, leading to no strain data for the low friction

condition. The inter-subject variability is high, whereas the data for a same subject is repeatable. The influence of friction on the compressive and tensile strain components is highlighted in Figure S3.

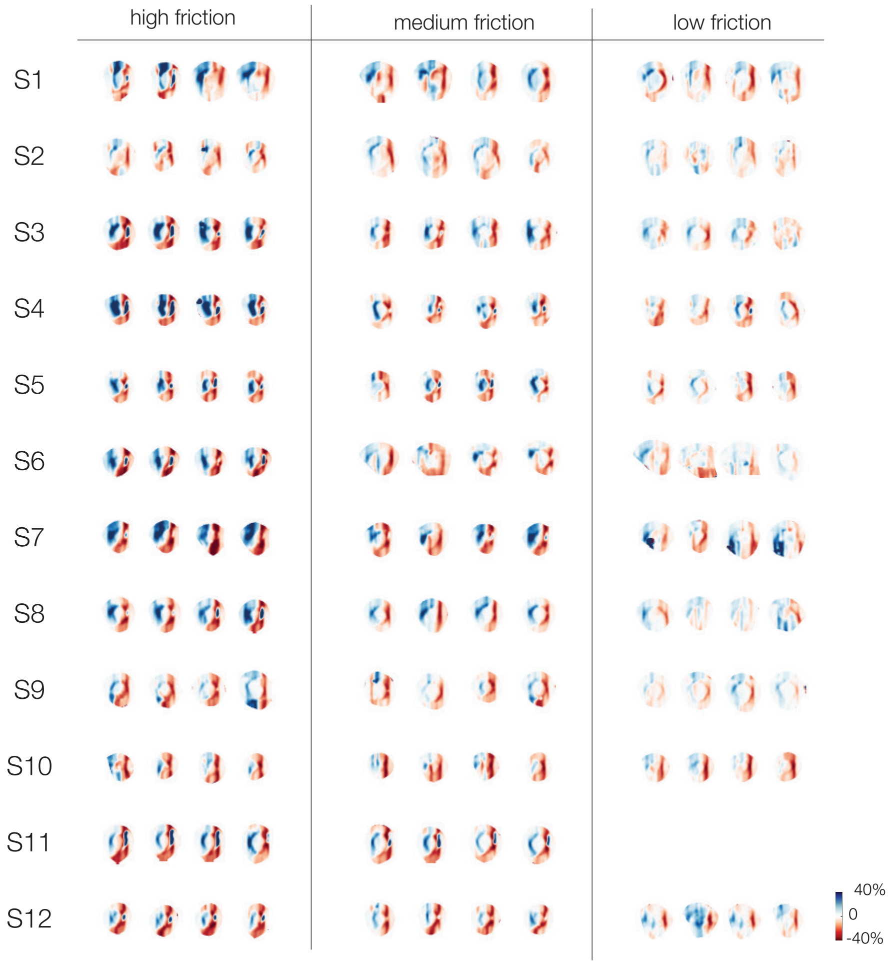

Figure S2: Strains for all subjects (S1 to S12) when the relative displacements between the finger and the plate is 6 mm.

2 Finite-difference mechanical model

The model is constructed with a bottom-up approach, using as few parameters to fit the observed phenomenon. The model is composed of a chain of massless elements maintained by springs. This chain can be assimilated to the external layer of the skin (the epidermis). To guarantee its shape, each element is linked

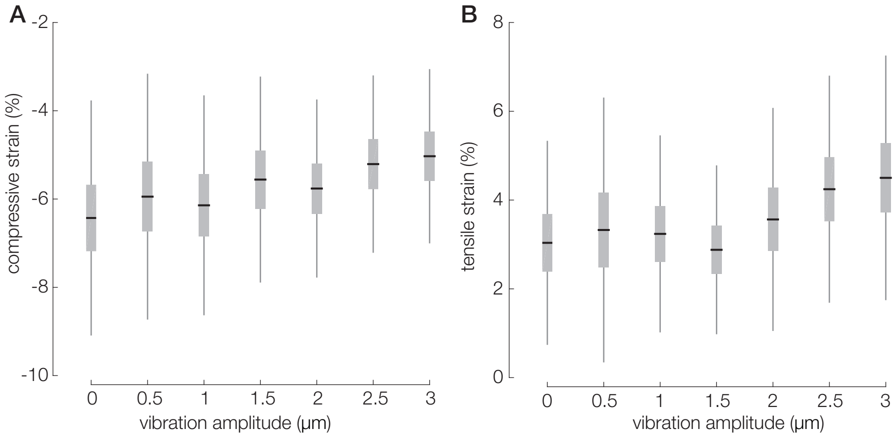

Figure S3: Median compressive strains (**A**) and tensile strains (**B**) as a function of the vibration amplitude when the relative displacement is 6 mm.

to a virtual bone by another springs which model the subcutaneous tissues. The both extreme elements are also attached to the bone to model the effect of the rigid nail. All spring connectivities are represented in figure S4A.

2.1 Dynamic equation

Each spring applies forces to its endpoint particles i proportional to the length deviation (linear elasticity), discounted by a damping term proportional to the rate of length change. Let F_i the forces acting on the particles i by all springs and dampers and U_i the displacement of the particles i. Then the internal force on each element i can be written as following (1).

$$F_i = -k_m(\mathrm{U}_{i-1} - 2\mathrm{U}_i + \mathrm{U}_{i+1}) - k_t(\mathrm{U}_i - \mathrm{U}_b) - \zeta \dot{\mathrm{U}}_i \tag{1}$$

where k_m is the stiffness of the external layer of the skin, k_t is the stiffness of the subcutaneous tissues and ζ is the damping coefficient.

Springs and dampers dependencies were then embedded in matrices **K** and **B** respectively. As each element is massless, the inertia is neglected (1). Then, the equation of motion can be written as following (2).

$$\mathbf{B}\dot{\mathrm{U}}(t) + \mathbf{K}(t)\mathrm{U}(t) + F_{ext}(t) = 0 \tag{2}$$

where U is the vector of normal and tangential displacements and F_{ext} is the vector of external forces. K is time dependent because it varies with the position of the elements.

The dynamic equation can be written in discrete time as following (3).

$$\mathbf{B}\left(\frac{\mathrm{U}(t+dt) - \mathrm{U}(t)}{dt}\right) + \mathbf{K}(t)\mathrm{U}(t) - F_{ext}(t) = 0 \tag{3}$$

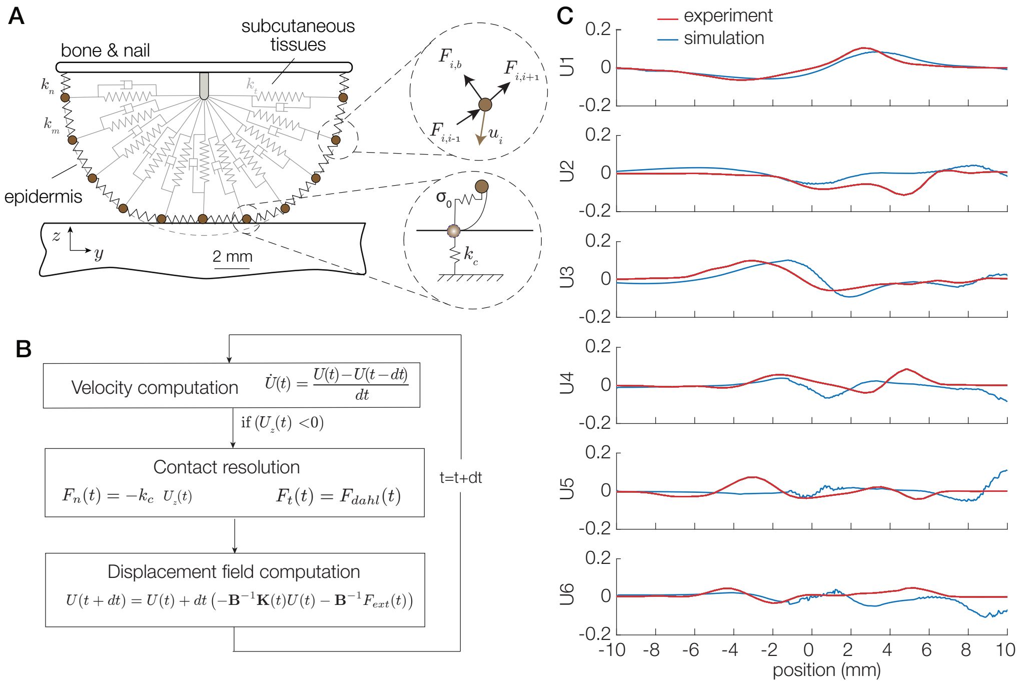

Figure S4: **A**. Sketch of the mechanical model with spring and dampers connectivities. **B**. Computational steps of the displacement vector. **C**. Comparison between the first 6 bases U obtained with the simulated and the experimental data.

$$\mathrm{U}(t + dt) = \mathrm{U}(t) + dt\left(-\mathbf{B}^{-1}\mathbf{K}(t)\mathrm{U}(t) - \mathbf{B}^{-1}F_{ext}(t)\right) \tag{4}$$

The algorithm follows the procedures steps showed in figure S4B. After resolving the contact, the forces were updated and displacements computed using Runge-Kutta algorithm at the fourth order.

2.2 Contact modeling

To model the finger contact with the surface, we use the penalty method, adding a high-stiffness spring k_c between one element in contact and the surface.

Modeling friction is one of the most challenging tasks because of its nonlinearity. Friction modeling affects a lot of areas, that is why numerous models were developed these last few years. Some of them are based on a spring-like relationship between frictional force and displacement. Dahl (2) proposed a model to compute the friction force in order to model pre-sliding displacement in control applications. In most general cases, the equation of the friction force can be written as in the following equation (5).

$$
\begin{aligned}
\frac{dF(x)}{dt} &= \frac{dF(x)}{dx}\frac{dx}{dt} \\
&= \sigma_0 \left|1 - \frac{F}{F_c} sign(\dot{x})\right|^n sign(1 - \frac{F}{F_c} sign(\dot{x}))\dot{x}
\end{aligned} \tag{5}
$$

where $F(x)$ is the friction force function, F_c is the coulomb friction force and σ_0 is the rest stiffness at equilibrium point $F = 0$, taken equal to $1e4$ here. n is a coefficient that codes how ductile or brittle the material is. Then $F(x)$ approaches the coulomb friction force F_c as long as $\dot{x} > 0$ and $-F_c$ when the direction of motion is reversed.

3 Dimensionality reduction

3.1 Set of bases obtained with simulation

Figure S4C shows the set of primitives obtained with the simulated (in blue) and the experimental (in red) strains computed for 7 friction conditions along the centerline of the finger. Both sets of bases are matching along the centerline, suggesting that the simulated and the experimental strains contained a similar information.

3.2 Choice of the method

The dimensionality reduction method was chosen based on a time-efficiency criterion. The technique must maximize the distance between classes while running in a reasonable amount of time. The distance was estimated with the Generalized Discrimination Value (GDV) Δ computed from the mean intra-class distances $\overline{d}$ and the mean inter-class distance d, Euclidean speaking:

$$
\Delta = \frac{1}{\sqrt{2}} \left(\frac{\overline{d}(C_{\mathrm{Sm}<0.5}) + \overline{d}(C_{\mathrm{Sm}>0.5})}{2} - d(C_{\mathrm{Sm}<0.5}, C_{\mathrm{Sm}>0.5}) \right) \tag{6}
$$

where the classes $C_{\mathrm{Sm}<0.5}$ and $C_{\mathrm{Sm}>0.5}$ correspond to a safety margin respectively lower and higher than 0.5. The GDV is always between 0 and -1, and a GDV close to -1 signals a high discriminability between the classes.

Fig. S5 shows the 2D-points in the space determined by the first 2 bases. The red and the blue color represent the safety margin lower and higher than 0.5 respectively. The GDV and the total classification time are shown above each plot. The singular value decomposition is the fastest method, keeping an acceptable Generalized Discrimination Value. It is interesting to note that even if the t-SNE algorithm has the lowest GDV, the mapping of the classification is not meaningful in terms of principal components.

Moreover, we found that the set of primitives extracted with the singular value decomposition is invariant to friction (Figure S6A), consistent with the human reflexive behavior with surfaces of various frictional strength. For those

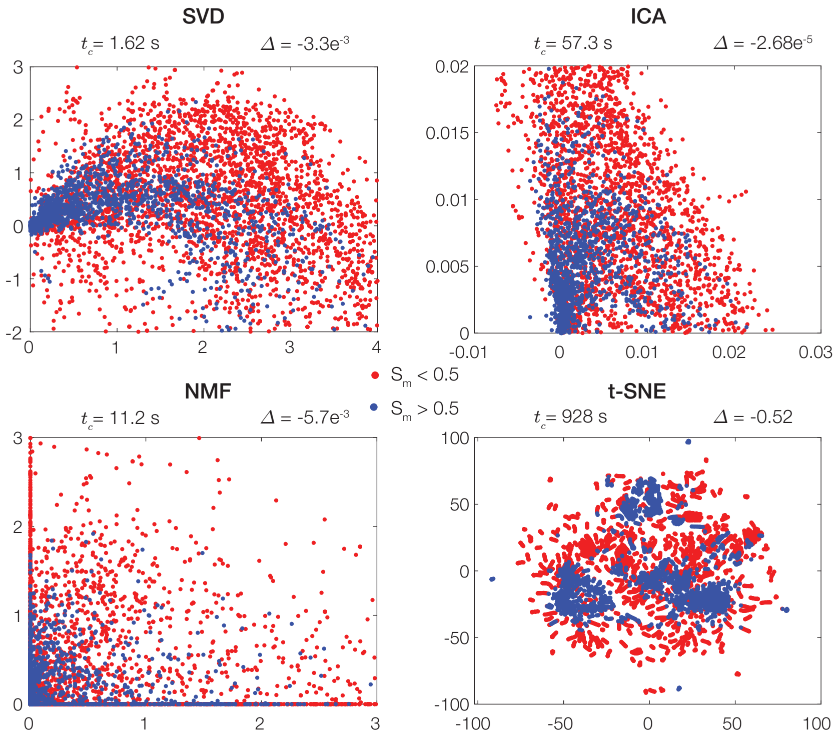

Figure S5: Comparison between dimensionality reduction methods. t_c and Δ stand for the classification time and the generalized discrimination value respectively.

reasons, we decided to use the singular value decomposition to reduce the dimensionality.

The data set matrix, $\mathbf{X}$, was approximated using a Singular Value Decomposition by the product of a $m \times r$ bases matrix, $\mathbf{U}$, with a $r \times r$ square matrix, $\mathbf{\Sigma}$, and a $r \times n$ matrix of activation weights, $\mathbf{V}$. The number of bases chosen for the approximation r is called the rank, $r \leq \min(m, n)$.

Overall, we find a trade-off between precision and compactness to choose the smallest number of bases to encode the safety margin with a sufficient accuracy. Kullback-Leibler divergence quantified the dissimilarity between $\epsilon(x, t)$ and $\hat{\epsilon}(x, t)$ as follows:

$$D_{KL}(\epsilon, \hat{\epsilon}) = \sum \left(\epsilon(x,t)\, ln \frac{\epsilon(x,t)}{\hat{\epsilon}(x,t)} - \epsilon(x,t) + \hat{\epsilon}(x,t) \right) \tag{7}$$

where $\hat{\epsilon}$ is the strains approximated using the r first bases. A small value of divergence means that u_i accurately captures the information contained in the strain data in all conditions. Divergence decreases with the truncation rank r

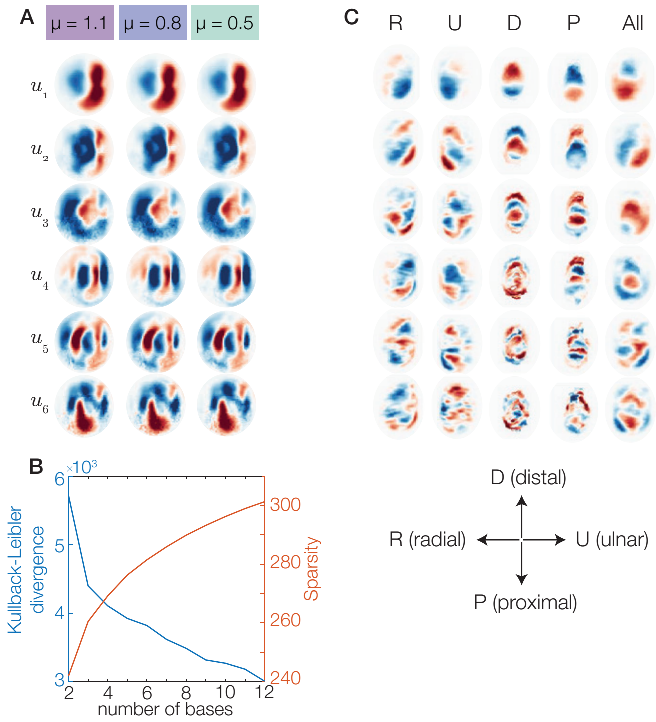

Figure S6: **A**. Primitives obtained are friction-invariant. **B**. Kullback-Leibler divergence and sparsity of the V matrix for an increasing number of bases. **C**. Primitives are direction-dependent.

and plateau for $r = 9$ (Figure S6B), denoting that a higher number of bases leads to more accurate estimate of the strain fields.

On the other hand, the number of bases that need to be activated, should also be restricted to promote compactness of the information. The compactness can be measured with the sparsity of the matrix V (expressed as the l_2 norm) for each value of the rank r, as follows:

$$S(V) = \sqrt{\sum_i v_i^2} \tag{8}$$

A higher number of bases increases the complexity of the computation since the V matrix become sparser (Figure S6B). A sparse matrix may result in a spreading of the main information on many primitives, which will be hard to capture in few milliseconds.

The method was finally applied to a dataset containing strains in the four directions (radial, ulnar, distal, proximal) (3). The set of bases obtained for the ulnar direction resembles the one we obtained in our study. However, we found direction-dependent primitives (Figure S6C), but interestingly the bases

obtained using the whole dataset (last column) are a combination of the directional primitives.

4 Safety margin prediction

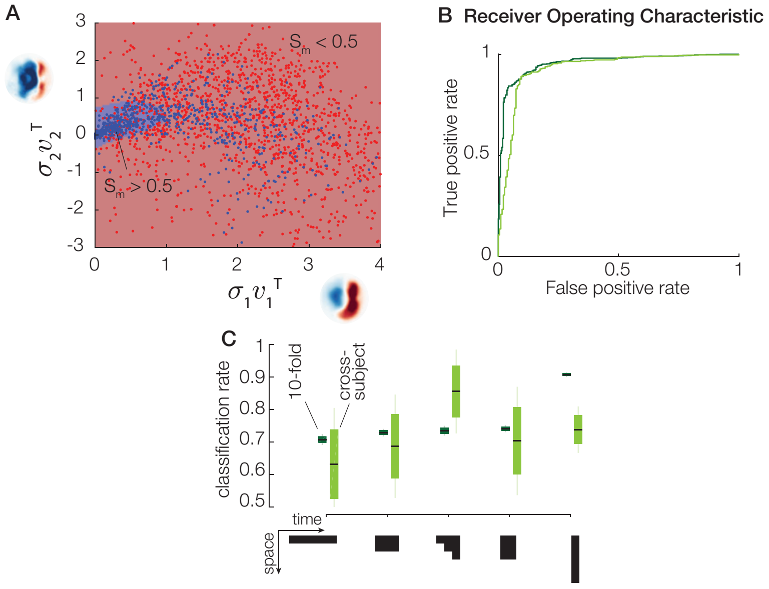

Figure S7: **A**. 2-dimensional mapping of 2 classes of the safety margin S_m (higher than 0.5 in blue and lower than 0.5 in red). **B**. Receiver Operating Characteristic of the both classifier (AUC = 0.9379 and 0.8353 for the ten-fold and the cross-subject respectively). **C**. Effect of time and space contributions to the classification rate from 6 temporal values to 6 spatial values (bases).

Figure S7A depicts the visual prediction map using the first two bases with the 10-fold classifier for 2 classes of safety margin: higher and lower than 0.5. When the weights $|\sigma_1 v_1^T|$ and $|\sigma_2 v_2^T|$ are higher than 1, the contact is close to full slippage ($S_m < 0.5$).

The receiver operating characteristics are plotted for the both classifiers in figure S7B. The curves exhibit areas under the curve higher than 0.8, meaning that both classifiers are able to distinguish between the 2 classes.

M1. Processed images of surface features displacements and computed strains for a high- and a low-friction condition.

[1] Michael Wiertlewski and Vincent Hayward. Mechanical behavior of the fingertip in the range of frequencies and displacements relevant to touch. *Journal of biomechanics*, 45(11):1869–1874, 2012.

[2] Philip R Dahl. Solid friction damping of mechanical vibrations. *AIAA journal*, 14(12):1675–1682, 1976.

[3] Benoit Delhaye, Allan Barrea, Benoni B Edin, Philippe Lefevre, and Jean-Louis Thonnard. Surface strain measurements of fingertip skin under shearing. *Journal of The Royal Society Interface*, 13(115):20150874, 2016.
